# Astaxanthin Inhibits Interleukin-6 Expression in Cerulein/Resistin-Stimulated Pancreatic Acinar Cells

**DOI:** 10.1155/2021/5587297

**Published:** 2021-07-24

**Authors:** Min Seung Kwak, Joo Weon Lim, Hyeyoung Kim

**Affiliations:** Department of Food and Nutrition, BK 21 FOUR, College of Human Ecology, Yonsei University, Seoul 03722, Republic of Korea

## Abstract

Acute pancreatitis is a common clinical condition with increasing the proinflammatory mediators, including interleukin-6 (IL-6). Obesity is a negative prognostic factor in acute pancreatitis. Obese patients with acute pancreatitis have a higher systemic inflammatory response rate. Levels of serum resistin, an adipocytokine secreted by fat tissues, increase with obesity. Cerulein, a cholecystokinin analog, induces calcium (Ca^2+^) overload, oxidative stress, and IL-6 expression in pancreatic acinar cells, which are hallmarks of acute pancreatitis. A recent study showed that resistin aggravates the expression of inflammatory cytokines in cerulein-stimulated pancreatic acinar cells. We aimed to investigate whether resistin amplifies cerulein-induced IL-6 expression and whether astaxanthin (ASX), an antioxidant carotenoid with anti-inflammatory properties, inhibits ceruelin/resistin-induced IL-6 expression in pancreatic acinar AR42J cells. We found that resistin enhanced intracellular Ca^2+^ levels, NADPH oxidase activity, intracellular reactive oxygen species (ROS) production, NF-*κ*B activity, and IL-6 expression in cerulein-stimulated AR42J cells, which were inhibited by ASX in a dose-dependent manner. The calcium chelator BAPTA-AM inhibited cerulein/resistin-induced NADPH oxidase activation and ROS production. Antioxidant N-acetyl cysteine (NAC) and ML171, a specific NADPH oxidase 1 inhibitor, suppressed cerulein/resistin-induced ROS production, NF-*κ*B activation, and IL-6 expression. In conclusion, ASX inhibits IL-6 expression, by reducing Ca^2+^ overload, NADPH oxidase-mediated ROS production, and NF-*κ*B activity in cerulein/resistin-stimulated pancreatic acinar cells. Consumption of ASX-rich foods could be beneficial for preventing or delaying the incidence of obesity-associated acute pancreatitis.

## 1. Introduction

Acute pancreatitis is a common inflammatory disorder, the incidence of which has been increasing over recent years [1]. Mild acute pancreatitis may be self-limiting and not requiring any treatment, but up to 20% of patients suffer a severe attack and between 15 and 20% of these will die [1, 2]. Despite improvements in treatment and critical care, severe acute pancreatitis is still associated with high mortality rates [2]. The earliest events in acute pancreatitis occur within acinar cells. Acinar cell injury early in acute pancreatitis leads to a local inflammatory reaction with increasing the proinflammatory mediators [3]. Among inflammatory mediators, serum level of interleukin-6 (IL-6) reflects the severity of acute pancreatitis [4] and acute lung injury associated with severe acute pancreatitis [5]. Thus, serum IL-6 level has been suggested to be an effective indicator for pancreatic lesions as well as the degree of inflammatory response [4]. Rao and Kunte [6] measured serum IL-6, IL-8, IL-10, and C-reactive protein (CRP) levels within 24 h of admission in forty patients of clinically predicted severe acute pancreatitis (SAP). They found that IL‐6 ≥ 28.90 pg/mL had a sensitivity of 62.86%, specificity of 80%, and positive predictive value (PPV) of 95.65%, demonstrating that IL-6 is the best among the tested biomarkers for predicting the progression to severe pancreatitis. Similarly, Inagaki et al. [7] showed that IL-6 levels correlate with disease severity in both experimental and human pancreatitis. Lesina et al. [8] observed serum levels of IL-6, CRP, alpha 2-plasmin inhibitor plasmin complex, IL-8, and soluble human E selectin at 5 and 72 h, respectively, after the onset of acute pancreatitis. There was a significant correlation between IL-6 at 5 h and both pancreatic secretory trypsin inhibitor (*r* = 0.85) and CRP (*r* = 0.94) at 72 h. They therefore concluded that IL-6 is a useful marker for assessment of the severity of acute pancreatitis in its early stages.

Obesity is associated with local and systemic complications in acute pancreatitis [9]. Obese patients with acute pancreatitis have a higher rate of a systemic inflammatory response and worse outcomes compared to nonobese patients [10]. Resistin is a cysteine-rich adipokine secreted by adipocytes and macrophages, which is associated with the development of obesity and diabetes [11]. Resistin is known to have a proinflammatory potential since serum resistin levels increase in parallel with C-reactive protein in acute pancreatitis [12]. Since serum resistin levels are high in the patients with acute pancreatitis [13], increased levels of resistin have been used as an early marker of inflammation in patients with acute pancreatitis [13].

Cerulein pancreatitis is one of the best characterized and widely used experimental models of acute pancreatitis. Supramaximal doses of cerulein, a cholecystokinin (CCK) analogue, result in experimental pancreatitis, which is characterized by dysregulation of the production and secretion of digestive enzymes, cytoplasmic vacuolization, death of acinar cells, edema formation, and infiltration of inflammatory cells into the pancreas [14].

High level of resistin induces inflammation and inflammation-related diseases like atherosclerosis and arthritis in animal and human studies [15, 16]. Resistin levels are increased in the pancreatic tissues of patients with acute pancreatitis, and the increased expression of resistin correlates with the severity of acute pancreatitis [17–19]. Jiang and Wang [20] demonstrated that resistin aggravates the expression of proinflammatory cytokines tumor necrosis factor-*α* (TNF-*α*) and IL-6 in cerulein-stimulated AR42J pancreatic acinar cells. Even though the mechanism underlying the effect of resistin in the aggravation of acute pancreatitis seems different from that of cerulein, these studies suggest that combined treatment of cerulein and resistin may augment severe acute pancreatic damage.

Cerulein binds to a CCK receptor, a G-protein-coupled receptor, that induces transient Ca^2+^ oscillation by activating phospholipase C (PLC) and subsequently inducing inositol 1,4,5-trisphosphate (IP_3_)-dependent Ca^2+^ release from the endoplasmic reticulum in pancreatic acinar cells [21, 22]. Resistin binds to Toll-like receptor 4 (TLR4) and initiates cytosolic Ca^2+^ overload by activating a pathway involving PLC and IP_3_ and mobilization of Ca^2+^ from intracellular Ca^2+^ stores [23, 24]. Resistin increased the expression of the proinflammatory chemokines through activation of NF-*κ*B in hepatic stellate cells [25] and pancreatic acinar cells [26]. These studies support the recent finding demonstrating that Ca^2+^ overload in pancreatic acinar cells can act as a key trigger in the pathogenesis of acute pancreatitis [27].

Oxidative stress is involved in the pathogenesis of acute pancreatitis [3, 4, 14]. NADPH oxidase is a membrane-bound enzyme complex that plays an important role in the production of endogenous reactive oxygen species (ROS) [28]. According to previous studies, Ca^2+^ modulates NADPH oxidase. Increased levels of intracellular Ca^2+^ stimulate ROS-generating system (NADPH oxidase, endoplasmic reticulum, and mitochondria) and ROS production [29]. ROS activate an oxidant-sensitive transcription factor, NF-*κ*B, and in turn induce inflammatory gene expression, leading to inflammatory responses in acute pancreatitis [14, 30]. Therefore, we can postulate the relation of Ca^2+^ overload, NADPH oxidase-mediated ROS generation, NF-*κ*B activation, and inflammatory cytokine expression in pancreatic acinar cells stimulated with cerulein and resistin.

Astaxanthin (ASX) is a xanthophyll carotenoid found in algae, yeast, and aquatic animals, such as salmons and lobsters. Due to its structure, which consists of a long backbone and hydroxyl and keto moieties at each polar end, ASX has especially powerful antioxidant capacity among other carotenoids [31]. Previously, we demonstrated that ASX inhibits *H*. *pylori*-induced oxidative stress, mitochondrial dysfunction, NF-*κ*B activation, and IL-8 expression in gastric epithelial cells [32]. Antioxidative and anti-inflammatory effects of ASX have been shown in diabetes, cardiovascular diseases, and neurodegenerative disorders [33]; however, the role of ASX in obesity-associated acute pancreatitis has not been investigated.

The present study is aimed at determining whether resistin amplifies cerulein-induced Ca^2+^ overload and NADPH oxidase-mediated ROS production and IL-6 expression in pancreatic acinar cells. Furthermore, we investigated whether ASX inhibits cerulein/resistin-induced IL-6 expression by suppressing Ca^2+^ overload and NADPH oxidase-mediated ROS production in AR42J cells.

## 2. Materials and Methods

### 2.1. Cell Line and Culture Condition

Rat pancreatic acinar AR42J cells (pancreatoma, CRL 1492) were obtained from American Type Culture Collection (Rockville, MD, USA) and maintained in Dulbecco's modified Eagle's medium (DMEM; Gibco, Grand Island, NY, USA) containing 10% fetal bovine serum and an antibiotic-antimycotic cocktail of 100 U/mL penicillin and 100 *μ*g/mL streptomycin. The cells were incubated at 37°g in a humidified atmosphere consisting of 95% air and 5% carbon dioxide. AR42J cells were grown to 80% confluence before use in the experiments.

### 2.2. Reagents

Resistin, cerulein, ASX, NADPH oxidase 1 inhibitor ML171, and the antioxidant N-acetyl cysteine (NAC) were purchased from Sigma-Aldrich. Cerulein was dissolved in PBS containing 0.1% BSA (final concentration 10^−4^ M), aliquoted, and stored at -20°C. ASX, ML171, and NAC were dissolved in dimethyl sulfoxide (DMSO). The intracellular calcium chelator BAPTA-AM was purchased from Abcam (Cambridge, UK) and dissolved in DMSO.

### 2.3. Experimental Protocol

To investigate the effect of ASX, the cells (2 × 10^5^/2 mL, 10 × 10^5^/10 mL) were pretreated with ASX (1 or 2 *μ*M) for 3 h. Then, the cells were prestimulated with resistin for 30 min prior to the addition of cerulein. Cells were stimulated with cerulein/resistin for 45 min (for NADPH oxidase activity, intracellular ROS levels, and ROS fluorescence imaging), 1 h (for NF-*κ*B DNA binding activity and immunofluorescence staining of NF-*κ*B p65), 6 h (for IL-6 mRNA expression), and 24 h (for IL-6 protein expression). To determine the involvement of NADPH oxidase, the cells were pretreated with ML171 (2 *μ*M), known as an NADPH oxidase 1 inhibitor, for 1 h before resistin/cerulein stimulation. To identify the antioxidant activity of ASX, the cells were pretreated with NAC (1 mM) for 1 h before cerulein/resistin stimulation. To ensure the involvement of Ca^2+^, the intracellular Ca^2+^ chelator BAPTA-AM (5 *μ*M) was added for 1 h before cerulein/resistin stimulation. Control cells received DMSO (less than 0.1%) alone instead of ASX, ML171, NAC, or BAPTA-AM.

Prior to the experiments on the effect of ASX on cerulein/resistin-induced alterations, time-course experiments were performed. The cells were treated with cerulein with or without resistin for 5 min (for Ca^2+^ level), 45 min (for ROS levels), 4 h (for NF-*κ*B activity), and 6 h (for IL-6 mRNA levels).

### 2.4. Preparation of Cell Extracts

For preparation of cell extracts, we followed the methods of Kim et al. [32]. Briefly, the cells were harvested by treatment with trypsin/EDTA, followed by centrifugation at 1000 × g for 5 min. The cell pellets were resuspended in lysis buffer containing 10 mM Tris pH 7.4, 15 mM NaCl, 1% NP-40, and protease inhibitor complex (Complete; Roche, Mannheim, Germany) and lysed by drawing the cells through a 1 mL syringe with several rapid strokes. The resulting mixture was incubated on ice for 30 min, followed by centrifugation at 13,000 × g for 15 min. The supernatants were collected and used as whole-cell extracts.

To prepare cytosol and membrane extracts, the cells were extracted in a homogenization buffer containing 10 mM Tris-HCl (pH 7.4), 50 mM NaCl, 1 mM ethylenediaminetetraacetic acid (EDTA), and protease inhibitor complex and centrifuged at 100,000 × g for 1 h. The supernatant was used as the cytosol extract. The pellets were resuspended on ice in lysis buffer containing 50 mM HEPES (pH 7.4), 150 mM NaCl, 1 mM EDTA, and 10% glycerol and used as membrane extracts.

To prepare nuclear extracts, the cells were extracted in a buffer containing 10 mM HEPES (pH 7.9), 10 mM KCl, 0.1 mM EDTA, 1.5 mM MgCl_2_, 0.05% NP-40, 1 mM DTT, and 0.5 mM phenylmethylsulfonyl fluoride (PMSF). The nuclear pellets were resuspended on ice in nuclear extraction buffer containing 20 mM HEPES (pH 7.9), 420 mM NaCl, 0.1 mM EDTA, 1.5 mM MgCl_2_, 25% glycerol, 1 mM DTT, and 0.5 mM PMSF and then centrifuged. The supernatants were used as nuclear extracts. Protein concentration was determined using the Bradford assay (Bio-Rad Laboratories, Hercules, CA, USA).

### 2.5. Measurement of Intracellular ROS Levels and Fluorescence Imaging

For the measurement of intracellular ROS, we followed the methods of Kim et al. [32]. The cells were stimulated with cerulein/resistin for 45 min and then loaded with 10 *μ*M dichlorofluorescein diacetate (DCF-DA; Sigma-Aldrich) for 30 min. The cells were then washed and scraped off with PBS. DCF fluorescence was measured (excitation at 495 nm and emission at 535 nm) using a Victor5 multilabel counter (PerkinElmer Life and Analytical Sciences, Boston, MA, USA).

For ROS fluorescence imaging, the cells on coverslips placed in 6-well plates were stimulated with cerulein/resistin for 45 min and then loaded with 10 *μ*M DCF-DA for 30 min. The cells were washed with PBS. Fluorescence images were acquired with a laser scanning confocal microscope (Zeiss LSM 880, Carl Zeiss Inc., Thornwood, NY, USA).

### 2.6. Measurement of NADPH Oxidase Activity

NADPH oxidase activity was measured using the lucigenin assay, as previously described [24]. The membrane and cytosolic fractions were prepared as described for the preparation of cell extracts. The assay was performed in 50 mM Tris-MES buffer (pH 7.0) containing 2 mM KCN, 10 *μ*M lucigenin, and 100 *μ*M NADPH as the substrate. The reaction was initiated by the addition of membrane fractions containing 10 *μ*g of protein. The photon emission was measured every 60 s for 5 min in a microtiter plate luminometer (Micro-Lumat LB 96 V luminometer, Berthold, NH, USA). NADPH oxidase activity in cytosolic extracts was also monitored and used as a negative control.

### 2.7. Measurement of Intracellular Ca^2+^ Level

For the measurement of intracellular Ca^2+^ levels, we followed the methods of Ku et al. [34]. Intracellular Ca^2+^ levels were measured using the cell-permeable fluo-4 AM dye (F14201; Thermo Fisher Scientific). The cells were plated in a 96-well plate (1.5 × 10^4^ cells/well) and then cultured overnight. The cells were loaded with fluo-4 by incubation with HEPES buffer (pH 7.4) containing 1 mM probenecid and 4 *μ*M fluo-4 for 1 h at 37°C and then stimulated with resistin/cerulein. Fluorescence was measured using a microplate reader (Molecular Devices, Sunnyvale, CA, USA), using an excitation wavelength of 494 nm and an emission wavelength of 525 nm. Ca^2+^ levels were expressed as ∆*F*/*F*_0__,_ where *F*_0_ is the resting background fluorescence, and ∆*F* is the fluorescence change over time after treatment with or without cerulein in the presence or absence of resistin. In another experiment, ∆*F* is the fluorescence change over time after treatment with or without cerulein/resistin in the presence or absence of AST.

### 2.8. Electrophoretic Mobility Shift Assay (EMSA) for NF-*κ*B-DNA Binding Activity

NF-*κ*B-DNA binding activity was determined as previously described [24]. Briefly, the NF-*κ*B gel shift oligonucleotide (5′-ACTTGAGGGGACTTTCCCAGGGC-3′) was radiolabeled using [^32^P]-dATP (Amersham Biosciences, Piscataway, NJ, USA) and T4 polynucleotide kinase (GIBCO, Grand Island, NY, USA). The radiolabeled oligonucleotide was separated from unconsumed [^32^P]-dATP using a Bio-Rad purification column (Bio-Rad Laboratories) and eluted with Tris-EDTA buffer. Nuclear extracts of the cells were incubated with the [^32^P]-labeled oligonucleotide in buffer containing 12% glycerol, 12 mM HEPES (pH 7.9), 1 mM EDTA, 1 mM DTT, 25 mM KCl, 5 mM MgCl_2_, and 0.04 *μ*g/mL poly[d(I-C)] at 20–22°C for 30 min. The samples were subjected to electrophoretic separation at 4°C on a nondenaturing 5% acrylamide gel. The gel was dried at 80°C for 2 h, followed by exposure at -80°C to a radiography film using an intensifying screen.

### 2.9. Immunofluorescence Staining for NF-*κ*B p65

The cells on coverslips placed in 6-well plates were pretreated with ASX, NAC, or ML171 and then stimulated with cerulein/resistin for 1 h. The cells were fixed in 4% formaldehyde. The fixed cells were permeabilized with 0.1% Triton X-100 in PBS for 5 min, blocked with 0.1% gelatin and 1% bovine serum albumin in PBS for 1 h, and then incubated for 1 h with the primary antibody for NF-*κ*B p65 (sc-7151, Santa Cruz Biotechnology, Dallas, TX, USA). After washing with PBS, the cells were incubated with rhodamine-conjugated mouse anti-rabbit IgG antibody (sc-2492, Santa Cruz Biotechnology) for 1 h. After removal of the secondary antibody, the cells were washed with PBS and covered with the antifade medium Vectashield containing 4′,6-diamidino-2-phenylindole (DAPI). The preparations were stored for 30 min to allow saturation with DAPI. The cells stained with rhodamine-conjugated antibody were examined with a laser scanning confocal microscope (Zeiss LSM 880) and photographed.

### 2.10. Real-Time PCR Analysis for IL-6 mRNA Expression

Total RNA was isolated using the TRI reagent (Molecular Research Center, Inc., Cincinnati, OH, USA). Total RNA was converted into cDNA by reverse transcription using a random hexamer and MuLV reverse transcriptase (Promega, Madison, WI, USA) and heated at 23°C for 10 min, 37°C for 60 min, and 95°C for 5 min. cDNA was used for real-time PCR with specific primers for IL-6 and *β*-actin. The sequences of the IL-6 (accession number M26745) primers used to produce the desired 242-bp PCR product were 5′-GCCCTTCAGGAACAGCTATGA-3′ (forward primer) and 5′- TGTCAACAACATCAGTCCCAAGA -3′ (reverse primer). For *β*-actin cDNA production, the desired 890 bp PCR product was obtained using the forward primer 5′- ACCAACTGGGACATGGAG -3′ and reverse primer 5′- GTCACGATCTTCATGAGGTAGTC-3′. For PCR amplification, the cDNA was amplified using the following conditions: 45 cycles of denaturation at 95°C for 30 s, annealing at 51°C for 30 s, and extension at 72°C for 30 s. During the first cycle, the denaturation step at 95°C was extended to 3 min. The *β*-actin gene was amplified in the same reaction to serve as a reference gene.

### 2.11. Enzyme-Linked Immunosorbent Assay (ELISA) for IL-6 Levels

Cells (2 × 10^5^ cells/well) were seeded in 6-well plates. The cells were pretreated with or without ASX for 3 h and then stimulated with cerulein/resistin for 24 h. The supernatants were centrifuged at 15,000 × g for 15 min at 4°C and collected to measure IL-6 levels. The level of IL-6 concentration in the medium was determined by enzyme-linked immunosorbent assay (ELISA) kits (Invitrogen Corporation, CA, USA) following the manufacturer's instructions.

### 2.12. Statistical Analysis

One-way ANOVA, followed by Newman-Keuls post hoc test, was used for statistical analysis. All data are reported as the mean ± standard error (SE) of three different experiments. For each experiment, the number of samples in each group was four (*n* = 4 per group). Differences were considered statistically significant at a *p* value of 0.05.

## 3. Results

### 3.1. Resistin Enhances Cerulein-Induced ROS Production, NADPH Oxidase Activation, and Ca^2+^ Increase in AR42J Cells

**Figure 1:**
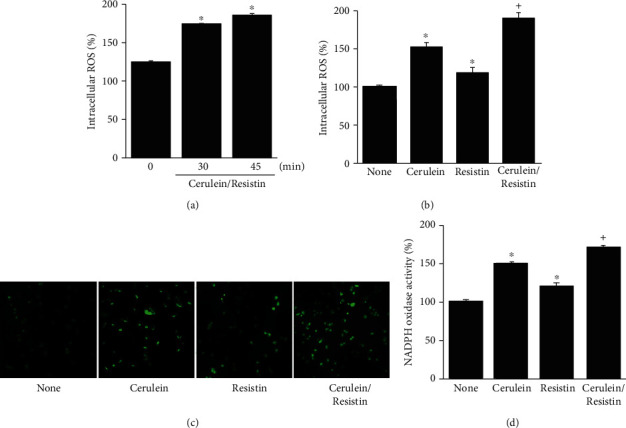
Resistin enhances cerulein-induced reactive oxygen species (ROS) production and NADPH oxidase activation in AR42J cells. (a) The cells were stimulated with cerulein/resistin for the indicated times. (b–d) The cells were stimulated with cerulein with or without resistin for 45 min. (a, b) Intracellular ROS levels were measured by dichlorofluorescein diacetate (DCF-DA) fluorescence. ROS levels were expressed as the relative increase. (c) Representative images of ROS-induced fluorescence response of DCF-DA. (d) NADPH oxidase activity in membrane fractions was measured by the lucigenin assay. Data are expressed as the mean ± S.E. of three different experiments. The value for cells without cerulein stimulation in the absence of resistin treatment (none) is set as 100%. ^∗^*p* < 0.05 vs. 0 min (a) or none (untreated cells; b, c); ^+^*p* < 0.05 vs. cerulein (cells treated with cerulein alone).

**Figure 2:**
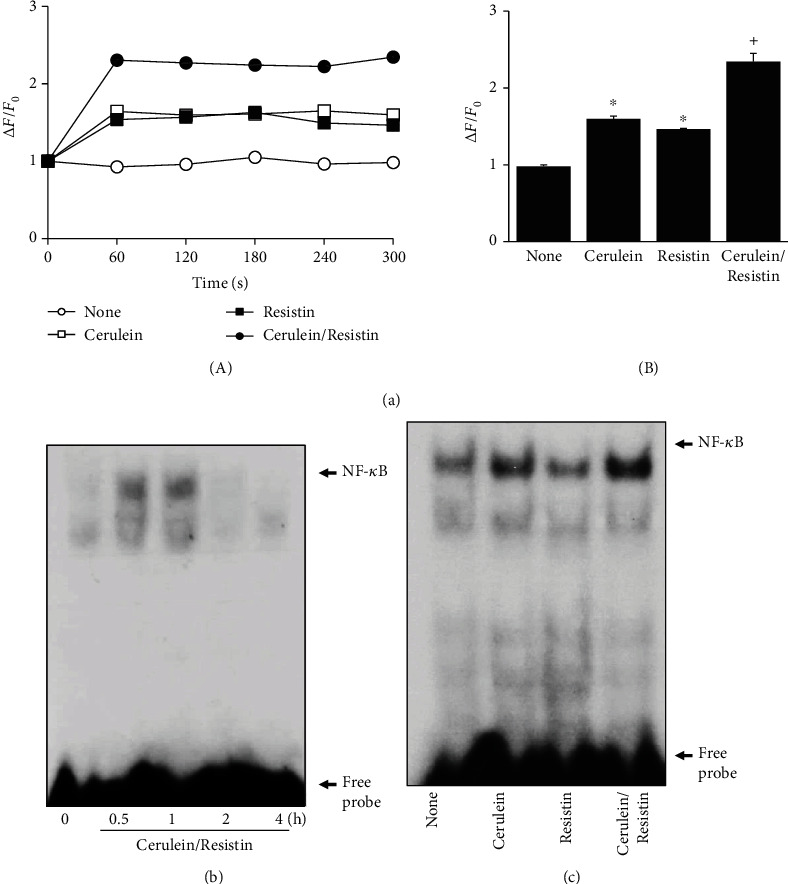
Resistin enhances cerulein-induced Ca^2+^ overload and NF-*κ*B activation in AR42J cells. (a) The cells were stimulated with cerulein in the presence or absence of resistin for the indicated times. Ca^2+^ level was determined by measuring the fluorescence changes of fluo-4 AM at excitation and emission wavelengths of 494 nm and 525 nm, respectively. Ca^2+^ levels were expressed as ∆*F*/*F*_0_, where *F*_0_ is the resting background fluorescence, and ∆*F* is fluorescence change over time after treatment with or without cerulein in the presence or absence of resistin. Fluorescence transient changes (obtained within 1-5 min) were plotted (A). (B) Ca^2+^ levels, expressed as ∆*F*/*F*_0_, of the cells. Data are expressed as the mean ± S.E. of three different experiments. Ca^2+^ level in none (untreated cells) was set as 1. ^∗^*p* < 0.05 vs. 0 min (a) or none (untreated cells; b, c); ^+^*p* < 0.05 vs. cerulein (cells treated with cerulein alone). (b) The cells were stimulated with cerulein/resistin for the indicated times. (c) The cells were stimulated with cerulein in the presence and absence of resistin for 1 h. NF-*κ*B-DNA binding activity was determined by electrophoretic mobility shift assay (EMSA).

Oxidative stress is regarded as a major causative factor in acute pancreatitis. To determine the effect of cerulein/resistin on ROS production, intracellular ROS levels were measured using DCF-DA fluorescence. Intracellular ROS levels were increased by cerulein/resistin, which reached a maximum at 45 min (Figure 1(a)). Therefore, a 45 min culture was used for further studies on the effect of resistin on ROS levels in cerulein-stimulated cells. As shown in Figures 1(b) and 1(c), resistin significantly enhanced intracellular ROS levels in cerulein-stimulated AR42J cells. To investigate the effect of resistin on cerulein-induced NADPH oxidase activation, NADPH oxidase activity was measured using the lucigenin assay. Resistin significantly enhanced NADPH oxidase activation in cerulein-stimulated AR42J cells (Figure 1(d)). Ca^2+^ overload in pancreatic acinar cells is a major pathogenic factor. To determine the effect of resistin on cerulein-induced Ca^2+^ overload, intracellular Ca^2+^ levels were monitored by transient fluorescence changes using fluo-4 AM (Figure 2(a)). Transient fluorescence changes (obtained within 1–5 min) were plotted. Ca^2+^ levels, expressed as ∆*F*/*F*_0_, were increased by cerulein or resistin. However, cerulein with resistin treatment showed higher Ca^2+^ levels than treatment with cerulein or resistin alone. To determine the effect of resistin on the cerulein-induced NF-*κ*B activation, NF-*κ*B-DNA binding activity was measured using EMSA. For the time-course experiment, NF-*κ*B activation was increased by cerulein/resistin at 30 min, which increased at 1 h (Figure 2(b)). After 1 h of culture, cerulein increased NF-*κ*B-DNA binding activity, which was potentiated by cotreatment with resistin in AR42J cells (Figure 2(c)).

### 3.2. Resistin Enhances Cerulein-Induced IL-6 Expression in AR42J Cells

**Figure 3:**
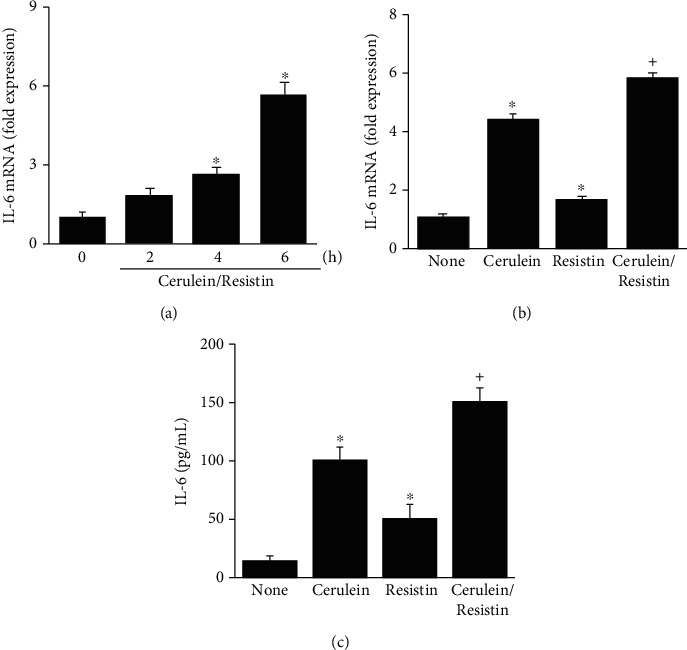
Resistin enhances the cerulein-induced IL-6 expression in AR42J cells. (a) The cells were stimulated with cerulein/resistin for the indicated times. (b, c) The cells were stimulated with cerulein in the presence and absence of resistin for 6 h (for mRNA level (b)) or 24 h (for protein level (c)). The mRNA level of IL-6 was determined by real-time PCR analysis and normalized to *β*-actin (a, b). The protein level of IL-6 in the media was determined by enzyme-linked immunosorbent assay (ELISA) (c). Data are expressed as the mean ± S.E. of three different experiments. ^∗^*p* < 0.05 vs. 0 min (a) or none (untreated cells; b, c); ^+^*p* < 0.05 vs. cerulein (cells treated with cerulein alone).

To investigate the effect of resistin on cerulein-induced IL-6 expression, IL-6 mRNA expression and IL-6 protein levels were determined by real-time PCR and ELISA, respectively. Cerulein/resistin increased IL-6 mRNA expression at 4 h, which was potentiated at 6 h (Figure 3(a)). Resistin significantly enhanced cerulein-induced IL-6 mRNA expression and IL-6 protein levels in the media (Figures 3(b) and 3(c)). The results show that cerulein with resistin treatment showed higher IL-6 expression than treatment with cerulein or resistin alone.

### 3.3. ASX Inhibits Cerulein/Resistin-Induced Increases in ROS and NADPH Oxidase Activity, Ca^2+^ Overload, NF-*κ*B Activation, and IL-6 Expression in AR42J Cells

**Figure 4:**
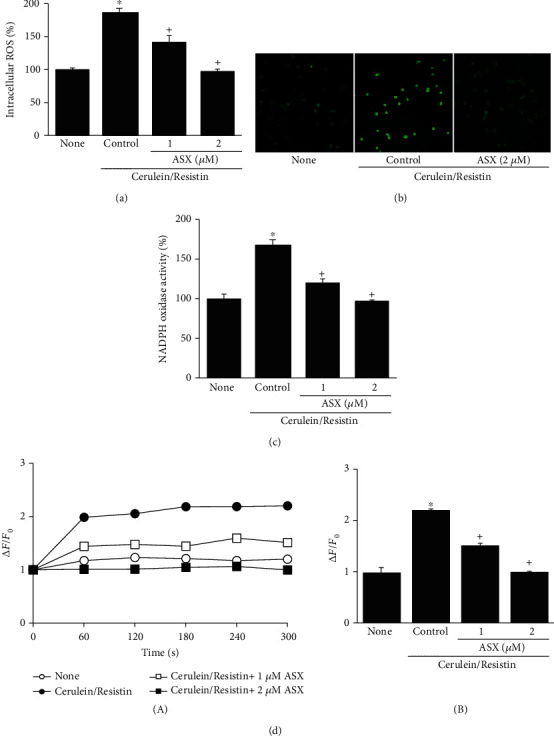
Astaxanthin (ASX) inhibits cerulein/resistin-induced increases in ROS, NADPH oxidase activity, and Ca^2+^ level in AR42J cells. The cells were pretreated with the indicated concentrations of ASX for 3 h and then stimulated with cerulein/resistin for 45 min (for ROS levels, ROS fluorescence imaging, and NADPH oxidase activity (a–c)), and for the indicated period (for Ca^2+^ level (d)). (a) Intracellular ROS levels were measured by dichlorofluorescein diacetate (DCF-DA) fluorescence. (b) Representative images of ROS-induced fluorescence response of DCF-DA. (c) NADPH oxidase activity was measured by the lucigenin assay. (d) Ca^2+^ level was determined by measuring the fluorescence changes of fluo-4 AM at excitation and emission wavelengths of 494 nm and 525 nm, respectively. Ca^2+^ levels were expressed as ∆*F*/*F*_0_, where *F*_0_ is the resting background fluorescence, and ∆*F* is fluorescence change over time after treatment with cerulein/resistin in the presence or absence of ASX. Fluorescence transient changes (obtained within 1-5 min) were plotted (A). (B) Ca^2+^ levels were expressed as ∆*F*/*F*_0_ of the cells. Data are expressed as the mean ± S.E. of three different experiments. ROS level, NADPH oxidase activity, and Ca^2+^ level in none (untreated cell) were set as 100 (a, c) or 1 (d). ^∗^*p* < 0.05 vs. none (untreated cells); ^+^*p* < 0.05 vs. cerulein (cells treated with cerulein alone).

**Figure 5:**
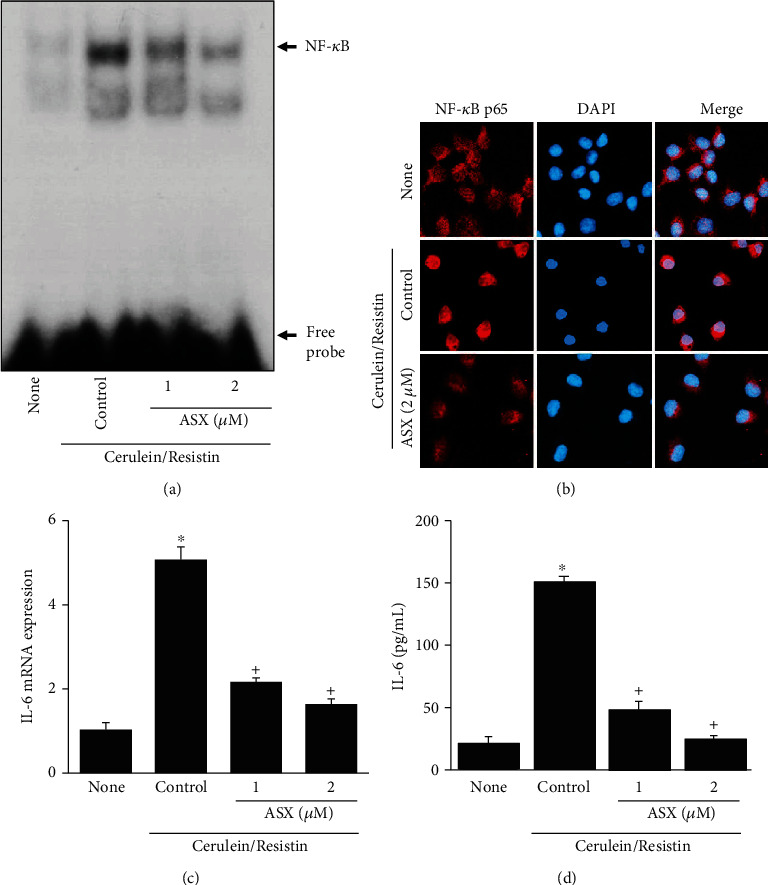
Astaxanthin (ASX) inhibits cerulein/resistin-induced NF-*κ*B activation and IL-6 expression in AR42J cells. The cells were pretreated with the indicated concentrations of ASX for 3 h and then stimulated with cerulein/resistin for 1 h (for NF-*κ*B-DNA binding activity and nuclear level of NF-*κ*B p65 by confocal images (a, b)), for 6 h (for IL-6 mRNA level (c)), and 24 h (for IL-6 protein level (d)). (a) NF-*κ*B-DNA binding activity was determined by electrophoretic mobility shift assay (EMSA). (b) The immunoreactive NF-*κ*B p65 was visualized using a rhodamine-conjugated mouse anti-rabbit IgG antibody (red) with DAPI counterstaining (blue) of the same field. (c) The mRNA expression level of IL-6 was determined by real-time PCR analysis and normalized to that of *β*-actin. (d) The protein level of IL-6 in the media was determined by enzyme-linked immunosorbent assay (ELISA). Data are expressed as the mean ± S.E. of three different experiments. ^∗^*p* < 0.05 vs. none (untreated cells); ^+^*p* < 0.05 vs. cerulein (cells treated with cerulein alone).

To investigate the effect of ASX on ROS production, the cells were stimulated with cerulein/resistin in the presence or absence of ASX, and intracellular ROS levels were measured using DCF-DA. ASX decreased the intracellular ROS levels in cerulein/resistin-stimulated cells (Figures 4(a) and 4(b)). The cerulein/resistin-induced increase in NADPH oxidase activity was inhibited by ASX in a dose-dependent manner (Figure 4(c)). ASX decreased Ca^2+^ overload in cerulein/resistin-stimulated AR42J cells (Figure 4(d)). In addition, ASX treatment dose dependently decreased NF-*κ*B activation in cerulein/resistin-stimulated AR42J cells (Figure 5(a)). We next investigated the effect of ASX on the nuclear level of NF-*κ*B p65 by using immunofluorescence staining and confocal analysis. As shown on Figure 5(b), NF-*κ*B p65 was exclusively localized in the cytoplasm in unstimulated AR42J cells with very little to none detected in the nuclei. In contrast, treatment of cerulein/resistin induced massive nuclear translocation of NF-*κ*B p65, which was inhibited by ASX (Figure 5(b)).

To investigate the effect of ASX on cerulein/resistin-induced IL-6 expression, AR42J cells were stimulated with cerulein/resistin in the presence or absence of ASX. ASX significantly reduced cerulein/resistin-induced IL-6 mRNA expression and IL-6 protein level in a dose-dependent manner in AR42J cells (Figures 5(c) and 5(d)).

### 3.4. NAC, ML171, or BAPTA-AM Inhibits Cerulein/Resistin-Induced ROS Production, NADPH Oxidase Activation, and Nuclear Translocation of NF-*κ*B p65 in AR42J Cells

**Figure 6:**
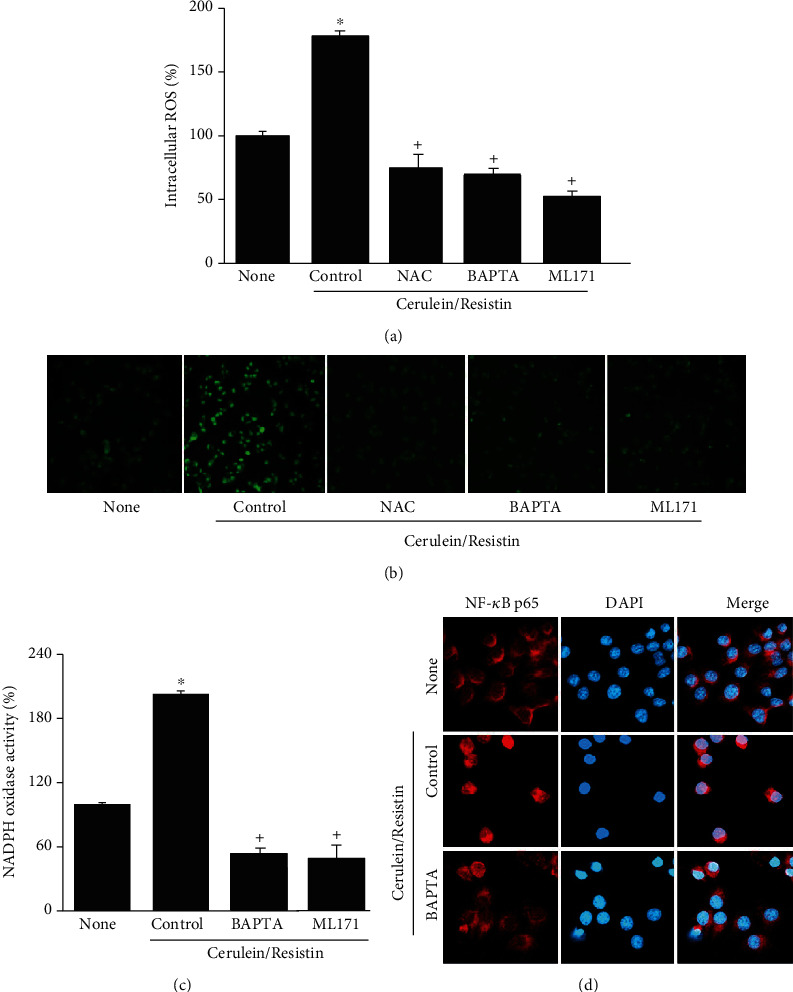
NAC, ML171, or BAPTA-AM inhibits cerulein/resistin-induced increases in ROS, NADPH oxidase activity, and nuclear translocation of NF-*κ*B p65 in AR42J cells. The cells were pretreated with NAC (1 mM) or ML171 (2 *μ*M) or BAPTA-AM (5 *μ*M) for 1 h and then stimulated with cerulein/resistin for 45 min (for ROS levels, ROS fluorescence imaging, and NADPH oxidase activity (a–c)), and for 1 h (for nuclear level of NF-*κ*B p65 by confocal images (d)). (a) Intracellular ROS levels were measured by dichlorofluorescein diacetate (DCF-DA) fluorescence. (b) Representative images of ROS-induced fluorescence response of DCF-DA. (c) NADPH oxidase activity in the membrane fraction was measured by the lucigenin assay. Data are expressed as the mean ± S.E. of three different experiments. ROS level and NADPH oxidase activity in none (untreated cells) were set as 100%. ^∗^*p* < 0.05 vs. none (untreated cells); ^+^*p* < 0.05 vs. cerulein (cells treated with cerulein alone). (d) The immunoreactive NF-*κ*B p65 was visualized using a rhodamine-conjugated mouse anti-rabbit IgG antibody (red) with DAPI counterstaining (blue) of the same field.

To examine whether cerulein/resistin-induced ROS production is linked to NADPH oxidase activation in AR42J cells, the cells were treated with an antioxidant NAC or a specific NADPH oxidase 1 inhibitor (ML171), before cerulein/resistin stimulation. Treatment with NAC or ML171 significantly suppressed the cerulein/resistin-induced increase in intracellular ROS levels and ROS fluorescence imaging (Figures 6(a) and 6(b)). Cerulein/resistin increased NADPH oxidase activity, which was significantly suppressed by treatment with ML171 (Figure 6(c)). These results suggest that cerulein/resistin increases ROS levels through NADPH oxidase activation in cells.

Because Ca^2+^ oscillation has been reported to stimulate the activity of NADPH oxidase, we investigated the effects of BAPTA-AM, a Ca^2+^ chelator, and observed that it prevented cerulein/resistin-induced increases in intracellular ROS levels and NADPH oxidase activity (Figures 6(a)–6(c)). To determine the relation between Ca^2+^ and NF-*κ*B activation, we investigated the effect of BAPTA-AM on the nuclear level of NF-*κ*B p65 in cerulein/resistin-stimulated cells, by using immunofluorescence staining and confocal analysis. The immunoreactive NF-*κ*B p65 was visualized using a rhodamine-conjugated mouse anti-rabbit IgG antibody with DAPI counterstaining of the same field. Cerulein/resistin increased the nuclear level of NF-*κ*B p65, which was reduced by BAPTA. These results show that Ca^2+^ plays a critical role on nuclear translocation of p65 in cerulein/resistin-stimulated cells (Figure 6(d)). Taken together, cerulein/resistin increases ROS levels and NF-*κ*B activation through NADPH oxidase activation and Ca^2+^ overload in AR42J cells.

### 3.5. NAC and ML171 Inhibit Cerulein/Resistin-Induced NF-*κ*B Activation and IL-6 Expression in AR42J Cells

**Figure 7:**
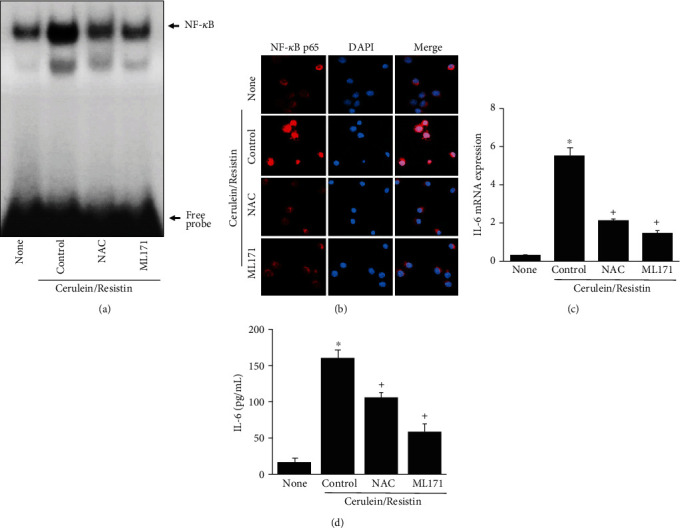
NAC and BAPTA-AM inhibits cerulein/resistin-induced NF-*κ*B activation and IL-6 expression in AR42J cells. The cells were pretreated with NAC (1 mM) or ML171 (2 *μ*M) for 1 h and then stimulated with cerulein/resistin for 1 h (for NF-*κ*B-DNA binding activity (a) and nuclear translocation of NF-*κ*B p65 (b)), for 6 h (for IL-6 mRNA level (c)), and 24 h (for IL-6 protein level (d)). (a) NF-*κ*B-DNA binding activity was determined by electrophoretic mobility shift assay (EMSA). (b) The immunoreactive NF-*κ*B p65 was visualized using a rhodamine-conjugated mouse anti-rabbit IgG antibody (red) with DAPI counterstaining (blue) of the same field. (c) The mRNA expression level of IL-6 was determined by real-time PCR analysis and normalized to that of *β*-actin. (d) The protein level of IL-6 in the media was determined by enzyme-linked immunosorbent assay (ELISA). Data are expressed as the mean ± S.E. of three different experiments. ^∗^*p* < 0.05 vs. none (untreated cells); ^+^*p* < 0.05 vs. cerulein (cells treated with cerulein alone).

To examine whether cerulein/resistin-induced NF-*κ*B activation and IL-6 expression are linked to ROS and NADPH oxidase activation in AR42J cells, the cells were treated with an antioxidant NAC or a specific NADPH oxidase 1 inhibitor (ML171), before cerulein/resistin stimulation. Treatment with NAC or ML171 significantly suppressed the cerulein/resistin-induced NF-*κ*B activation (Figure 7(a)), nuclear translocation of NF-*κ*B p65 (Figure 7(b)), and IL-6 expression at mRNA and protein levels (Figures 7(c) and 7(d)). These results suggest that NADPH oxidase-mediated production of ROS may induce NF-*κ*B activation and IL-6 expression in cerulein/resistin-stimulated AR42J cells.

## 4. Discussion

Obesity is rapidly spreading worldwide, and it is a recognized health hazard. Pancreatologists have identified obesity as a risk factor for poor outcomes in patients with acute pancreatitis. Meta-analysis indicates that obesity exerts adverse effects on the development of severe acute pancreatitis, accompanied by local and systemic complications [35]. Resistin is an obesity-associated adipocytokine. Higher circulating levels of resistin were shown in hospitalized patients with severe acute pancreatitis than healthy subjects [36, 37]. These studies demonstrate that resistin may contribute to inflammatory response and may be useful as an early marker of inflammation in acute pancreatitis.

We previously demonstrated that stimulation of the AR42J cells with cerulein induces proinflammatory cytokine expression and results in the complete development of an *in vitro* model of acute pancreatitis [38–43]. Here, we found that resistin amplified the expression of the proinflammatory cytokine IL-6 in cerulein-stimulated AR42J cells. Yu et al. [44] showed that the levels of resistin are significantly increased in acute pancreatitis patients with persistent organ failure, in both the overweight and the nonoverweight subgroups. Resistin demonstrated similar accuracy with the Acute Physiology and Chronic Health Evaluation II (APACHE-II) score in predicting persistent organ failure in the overweight and nonoverweight subgroups. Taken together, overproduction of resistin and accompanying inflammatory response may lead to the aggravation of severe acute pancreatitis, possibly with the accompanying organ failure.

Recently, Peres et al. [45] showed that obesity causes the deficiency of peroxisome proliferator-activated receptor-*γ* (PPAR*γ*) coactivator 1*α* (PGC-1*α*), a transcriptional coactivator and master regulator of mitochondrial biogenesis in the pancreas. Since PGC-1*α* acts as selective repressor of NF-*κ*B towards IL-6 in pancreas, PGC-1*α* deficiency markedly enhanced NF-*κ*B-mediated upregulation of IL-6 in pancreas, leading to a severe pancreatitis. It is necessary to determine the effect of resistin on pancreatic PGC-1*α* in the pathogenesis of acute pancreatitis for the further study.

Acute pancreatitis is induced mainly by the overproduction of ROS. NADPH oxidase is a major source of ROS in pancreatitis models [14, 46]. We previously demonstrated that cerulein increased ROS production through NADPH oxidase activation in AR42J cells in a Ca^2+^-dependent manner [47]. In the present study, resistin significantly enhanced intracellular Ca^2+^ levels, NADPH oxidase activation, and ROS production in cerulein-stimulated AR42J cells. Moreover, cerulein/resistin-induced increases in intracellular ROS levels and NADPH oxidase activity were decreased by BAPTA-AM, a Ca^2+^ chelator. The results showed that intracellular Ca^2+^ mediates cerulein/resistin-induced NADPH oxidase activation and ROS production.

As described previously, binding of cerulean to CCK receptor initiates transient Ca^2+^ oscillation by activating PLC and inducing IP_3_-dependent Ca^2+^ release from the endoplasmic reticulum in pancreatic acinar cells [21, 22]. The receptor for resistin is known as TLR4 [23, 24]. Crosstalk with TLR4, Ca^2+^, and NADPH oxidase has been reported in human myeloid and epithelial cells [48] and the immune system, including phagocytes [49]. Park et al. reported the direct interaction of NADPH oxidase (NOX4) through TLR4 in kidney epithelial cells (HEK293T) and U937 monocytic cells [50]. TLR4 physically interacts with the C-terminus of NOX4, and this interaction is important for lipopolysaccharide-mediated NF-*κ*B activation in human aortic endothelial cells [51]. Resistin binds to TLR4 and initiates cytosolic Ca^2+^ by activating a pathway involving PLC and IP_3_ [23, 24].

From the present results, we can conclude that cerulein or resistin increases NADPH oxidase activity via the Ca^2+^-dependent pathway. Combination of cerulein and resistin potentiates intracellular Ca^2+^ level and induces NADPH oxidase-mediated ROS generation in pancreatic acinar cells. Moreover, the NADPH oxidase 1 inhibitor ML171 blocked cerulein/resistin-induced ROS production, NF-*κ*B activation, and IL-6 expression in the present study. Therefore, Ca^2+^-mediated NADPH oxidase activation is involved in cerulein/resistin-induced intracellular ROS production, NF-*κ*B activation, and IL-6 expression in pancreatic acinar cells. Further study should be performed to determine cell images for Ca^2+^ to investigate the role of Ca^2+^ on inflammatory signaling in cerulein/resistin-stimulated pancreatic acinar cells.

The antioxidant NAC inhibited cerulein/resistin-induced NF-*κ*B activation and IL-6 expression in AR42J cells. These results indicate that NADPH oxidase-mediated ROS may induce IL-6 expression through NF-*κ*B activation in cerulein/resistin-stimulated AR42J cells. We investigated the effect of BAPTA-AM, a Ca^2+^ chelator, and observed that it prevented cerulein/resistin-induced increases in intracellular ROS levels and NADPH oxidase activity. These results indicate that Ca^2+^ is upstream activator for NADPH oxidase in pancreatic acinar cells stimulated with cerulean/resistin. We previously showed that lycopene inhibits oxidative stress-mediated expression of IL-6 by suppressing the NADPH oxidase activity in ethanol/palmitoleic acid-stimulated pancreatic acinar AR42J cells [52]. Our previous and present findings suggest the involvement of NADPH oxidase on the pathogenesis of acute pancreatitis.

The pancreatic acinar cell is the functional unit of the exocrine pancreas. It synthesizes, stores, and secretes digestive enzymes. Disorders in these functions often lead to pancreatitis, an inflammatory disease of the pancreas. Hypersecretion of amylase and lipase, intracellular zymogen activation, oxidant stress, and cytokine secretion contribute to acinar cell injury. Therefore, function assays for pancreatic acinar cells include assays of intracellular zymogen activation, oxidant stress, inflammatory mediators, and digestive enzymes in acinar cells. During acute pancreatitis, injured pancreatic acinar cells release a series of proinflammatory mediators such as IL-6, which promote the recruitment and activation of immune cells. Previously, we found that vacuolar ATPase activation is essential for zymogen activation in pancreatic acinar cells stimulated with cerulein [39]. In the present study, the cerulein/resistin-stimulated IL-6 expression is used as a biomarker for pancreatic acinar cell damage.

Chao et al. [53] demonstrated that blockade of cerulein-induced IL-6 accelerates acinar cell apoptosis and attenuates experimental acute pancreatitis in vivo. Therefore, cerulein/resistin-stimulated IL-6 expression may prevent pancreatic acinar cell apoptosis. Further studies are necessary to determine whether cerulein/resistin activates zymogen and increases digestive enzyme secretion in pancreas, which may contribute to development of severe acute pancreatitis.

**Figure 8:**
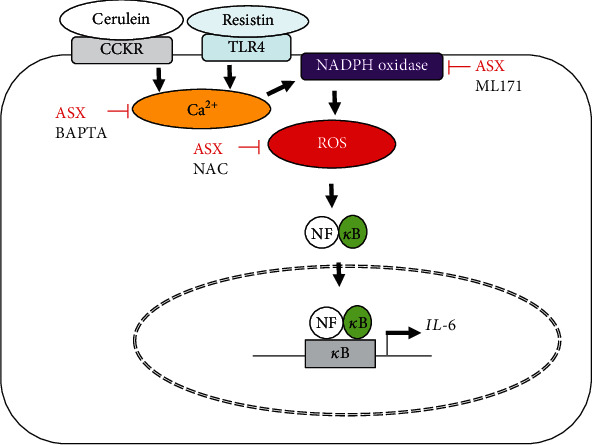
The proposed mechanism by which astaxanthin (ASX) inhibits interleukin-6 (IL-6) expression in cerulein/resistin-stimulated pancreatic acinar cells. Binding of cerulein to cholecystokinin receptor (CCKR) increases intracellular Ca^2+^ level while binding of resistin to Toll-like receptor 4 (TLR4) initiates Ca^2+^ overload. High level of Ca^2+^ activates NADPH oxidase to produce reactive oxygen species (ROS). ROS induce NF-*κ*B activation and the expression of IL-6 in pancreatic acinar AR42J cells. ASX reduces Ca^2+^ overload and inhibits NADPH oxidase-mediated ROS production, NF-*κ*B activation, and IL-6 expression. The calcium chelator BAPTA-AM, an antioxidant N-acetyl cysteine (NAC), and ML171, a specific NADPH oxidase 1 inhibitor, suppress cerulein/resistin-induced ROS production, NF-*κ*B activation, and IL-6 expression in AR42J cells.

In this study, ASX suppressed NADPH oxidase activation by reducing Ca^2+^ overload and, thus, inhibited ROS production, NF-*κ*B activation, and IL-6 expression in cerulein/resistin-stimulated pancreatic acinar cells (Figure 8). Therefore, ASX has an inhibitory effect on cerulein/resistin-induced NF-*κ*B activation and IL-6 expression by suppressing the increase in Ca^2+^ and NADPH oxidase activity in pancreatic acinar AR42J cells.

## 5. Conclusions

The results from the present study demonstrate (1) that resistin amplifies cerulein-induced IL-6 expression in pancreatic acinar cells by increasing Ca^2+^ level, NADPH oxidase-mediated ROS production, and NF-*κ*B activation and (2) that ASX inhibits cerulein/resistin-induced IL-6 expression in pancreatic acinar cells by suppressing Ca^2+^ overload and NADPH oxidase-mediated ROS production and NF-*κ*B activation. Therefore, consumption of ASX-rich foods may be beneficial for preventing the development of obesity-associated acute pancreatitis.

## Data Availability

The data used to support the findings of this study are included within the article.
